# How Does Digital Integration Influence the Mental Health of Low-Income Populations?

**DOI:** 10.3390/healthcare12242593

**Published:** 2024-12-23

**Authors:** Xiaoli Wen, Beihai Tian

**Affiliations:** School of Humanities & Social Sciences, Huazhong Agricultural University, No. 1 Shizishan Street, Wuhan 430070, China; 105102024163@mail.hzau.edu.cn

**Keywords:** mental health, digital integration, social participation, low-income populations

## Abstract

**Background:** In recent years, the growing prevalence of digital technology has transformed every aspect of life, including mental health care and support. Digital integration—referring to both access to digital technology and the utilization of digital tools in daily life—has the potential to alleviate existing health inequalities, a phenomenon that has been labeled the ‘digital divide’. It is, therefore, imperative to gain an understanding of the mental health dynamics of vulnerable groups in the context of the digital age if we are to develop effective policies and interventions. **Objectives:** The present study aimed to expand the existing knowledge base on the impact of digital integration on the mental health of low-income populations, with a specific focus on its association with social participation and the contextual variations observed between urban and rural settings. **Methods:** A total of 930 Chinese urban and rural low-income residents (≥18 years old) were included in the study using data from the Comprehensive Social Survey of Urban and Rural Low-Income Populations in Hubei Province, China, 2022. Multiple linear regression, generalized propensity score matching, causal step regression, and bootstrap methods were adopted to assess the direct impact of digital integration on the mental health of low-income populations, as well as to test the mediating role of social participation. **Results:** Digital integration was positively associated with the mental health of low-income populations, particularly reducing symptoms of anxiety and depression among low-income individuals, which remains robust even after accounting for potential selective bias. Social participation played a significant mediating role in the relationship between digital integration and the mental health of low-income populations. Heterogeneity analyses indicated that while digital integration has been demonstrated to markedly enhance mental health outcomes among urban low-income groups, this effect has not been observed in rural low-income populations. **Conclusions:** The present study contributes to the growing understanding of digital integration as a pathway to reducing mental health inequalities. It is imperative that targeted interventions be implemented to enhance digital integration and, in turn, improve mental health outcomes in low-income communities, particularly in rural areas, where the impact is less discernible.

## 1. Introduction

Mental health disorders are a significant global health challenge, and low-income populations are disproportionately affected by mental health issues [1]. These individuals often experience higher levels of psychological distress, depression, anxiety, and other mental health conditions due to factors such as financial strain, social instability, and limited access to healthcare services [2]. This issue is not exclusive to China; rather, it is a pervasive phenomenon in numerous low-income and developing countries. For example, in Subsaharan Africa and certain regions of South Asia, financial deprivation, social exclusion, and limited access to mental healthcare contribute to similar mental health disparities [3,4]. In China, however, the circumstances are particularly intricate due to the country’s accelerated urbanization, a considerable disparity between urban and rural areas, and the challenges associated with digital access [5].

Those in low-income populations in both urban and rural China encounter considerable obstacles to mental healthcare, which are intensified by the digital divide. Despite the fact that urban areas have comparatively greater access to healthcare services and digital technologies, rural populations frequently encounter inadequate infrastructure, limited digital literacy, and unreliable internet access [6]. These factors can impede individuals from fully utilizing digital tools, such as social media, mobile health apps, and online services, despite their potential to enhance mental health outcomes. Similar challenges are observed in other countries, such as India, where rural populations also face limitations in digital connectivity, low levels of digital literacy, and socio-economic disadvantages that impede access to both healthcare and digital health resources [7].

It is increasingly evident that digital technology exerts a significant influence on social participation and mental health outcomes on a global scale [8]. In numerous high-income countries, digital tools have been shown to be effective in addressing mental health needs by facilitating remote access to care and support. Nevertheless, in low-income or developing countries, including China, the digital divide continues to represent a substantial barrier to the realization of these benefits [9]. While certain technologies, such as telemedicine and online therapy, have demonstrated positive impacts on mental health in countries like the United States and the United Kingdom [10], their effectiveness in low-income settings is contingent upon infrastructure, affordability, and digital literacy [11]. Furthermore, social media, while facilitating connections, can occasionally exacerbate mental health issues, contributing to the development of anxiety and depression, particularly among vulnerable populations [12]. The impact of digital technology on mental health is, therefore, not inherently positive or negative; rather, it depends on how the technology is used, the specific context in which it is applied, and the type of technology involved.

Notwithstanding the global acknowledgement of the digital divide, research investigating the influence of digital integration on the mental well-being of low-income populations, particularly in rural and urban settings, remains scarce. Despite the eradication of absolute poverty in China in 2020, a considerable proportion of the population continues to live below internationally accepted relative poverty standards. These include the widely used benchmarks of 50% or 60% of the median disposable income per capita, common in European countries, and the World Bank’s poverty line of USD 6.85 per person per day for middle- and high-income countries. By focusing on both urban and rural low-income populations in China, this study will not only elucidate domestic disparities but also contribute to global discourses on the potential mitigating or exacerbating effects of digital access on mental health issues in low-income populations worldwide. The findings have the potential to inform policy and intervention strategies designed to reduce health inequalities and enhance access to digital resources, offering insights that may be relevant for other countries facing similar challenges.

## 2. Literature Review

### 2.1. The Digital Integration of Low-Income Populations

The digital divide has remained a persistent issue, with segments of the population, particularly those in low-income communities, facing restricted access to digital resources such as broadband internet, computers, and technological literacy [13]. This divide can serve to exacerbate existing disparities in areas such as education, healthcare, and employment opportunities, thereby perpetuating cycles of poverty [14]. A number of studies have identified the multifaceted barriers encountered by those with low incomes in accessing and utilizing digital technologies. One of the primary barriers is economic in nature, whereby the cost of digital devices and internet services is a significant financial burden for many individuals and families. To illustrate, the cost of smartphones, computers, or reliable broadband connections can be a significant barrier, particularly in rural or economically disadvantaged areas [15]. This lack of access precludes individuals from participating fully in contemporary society, where digital tools are indispensable for education, employment, and social interaction [16].

In addition to financial barriers, a dearth of digital literacy is another considerable challenge. Low-income individuals often have limited exposure to technology and may lack the requisite digital literacy skills to navigate the digital world effectively [17]. This lack of familiarity can impede their ability to utilize available digital tools for personal, professional, or educational purposes [18]. Furthermore, the physical infrastructure also plays a role in digital exclusion. In rural areas, unreliable internet connectivity, poor broadband infrastructure, and limited access to high-speed networks serve to exacerbate the digital divide [19]. This can further hinder the utilization of digital services, including telehealth, online education, and remote work opportunities, thereby limiting access to resources that could alleviate poverty [20].

### 2.2. Digital Integration and Mental Health

The advent of the digital age has transformed nearly every aspect of human life, including the realm of mental health care and support [21]. Digital mental health (DMH), which refers to the use of digital technologies such as the internet, mobile apps, telemedicine, and social media in mental health interventions, has shown promise in improving access to mental health care [22], optimizing treatment outcomes, and mitigating the stigma associated with mental health concerns [23]. A principal area of investigation in the field of research in digital integration and mental health is the development and evaluation of DMH interventions. These interventions, which encompass mobile applications, online therapy platforms, virtual reality (VR) therapy, and digital self-help tools, are devised with the objective of optimizing mental healthcare delivery [24], particularly in regions with limited access to conventional face-to-face therapy [25]. Research has consistently shown that digital interventions can be effective for treating a variety of mental health conditions, including depression, anxiety, post-traumatic stress disorder (PTSD), and stress-related disorders [26]. Furthermore, few studies have demonstrated the potential of digital technology overcoming several identified barriers to quality care to reach traditionally underserved and marginalized populations including those facing challenges related to access, cost, transportation, and stigma [27]. Research by Ramos and Chavira reviewed the empirical support of different digital mental health interventions (DMHIs) with racial and ethnic minorities and showed the ability of technology-based interventions to make services available for traditionally underserved populations such as racial and ethnic minorities (R&EM) and to reduce longstanding disparities in care [28]. However, challenges related to patient privacy, technological literacy, and the need for appropriate clinical training for providers remain essential considerations [29].

In recent times, social media and online communities have become indispensable sources of social support, particularly for individuals experiencing mental health difficulties. Research has explored the potential of digital social networks and online forums to provide peer support, reduce isolation, and promote mental health awareness [30]. Intersections between digital technology engagement and mental health have become increasingly salient during the COVID-19 pandemic, and the utilization of digital technology to facilitate social connections and mitigate the adverse effects of the pandemic has assumed heightened significance [31]. According to research by Marciano et al., the experience of loneliness and stress was mitigated by the presence of positive, mutually beneficial online relationships characterized by one-to-one communication and self-disclosure, and the online sharing of positive and humorous experiences appeared to have a beneficial effect on the emotional state of the participants during the COVID-19 pandemic [32]. Nevertheless, concerns about the potential negative effects of social media on mental health have been raised in several studies. Research by Twenge et al. showed that excessive use of new media, including social media and electronic devices such as smartphones, has been linked to an increased risk of depression and may be a contributing factor to the rising rates of suicide among adolescents [33]. In a more recent study by Smith et al., using cross-sectional data from a representative sample of the UK public, results indicated that a positive correlation was observed between screen time per day in hours and poor mental health, particularly in women and adults aged 35–64 years during the COVID-19 pandemic [34]. These findings suggest that the impact of digital social support is nuanced and depends on how individuals interact with online platforms.

## 3. Aims of the Current Study

The majority of studies have concentrated on the impact of digital technologies on mental health promotion, with a particular focus on accessibility, affordability, and effectiveness. Nevertheless, there has been a dearth of research examining the role of digital technologies in social engagement, despite the growing recognition of the importance of social interaction in mental health. In contrast to existing studies that concentrate exclusively on internet or social media utilization, the incorporation of the concept of digital integration in this study facilitates a more comprehensive evaluation of the capacity of low-income populations to access and employ digital technologies in daily life, as well as the favorable impact of these technologies on psychological well-being. It is notable that existing research has not adequately addressed the mental health-promoting effects of digital integration for low-income populations, the majority of research remains limited to young populations (adolescents, college students, children, etc.) and elderly populations, and most research fails to provide an in-depth analysis of the mechanisms through which digital integration affects mental health. The present study aims to address these gaps by examining the intersection of digital integration and mental health in low-income populations. Specifically, the study explores how the integration of digital technologies—such as mobile apps, social media, and other online services—can positively influence mental health outcomes. It examines the ways in which digital integration can promote mental health, including the alleviation of depressive and anxiety disorders, while also considering the potential benefits of digital technologies in improving mental well-being. Through this investigation, we seek to highlight the urgent need for policies and initiatives that bridge the digital divide and promote digital literacy to ensure that everyone, regardless of their economic status, has the opportunity to access mental health resources and support networks that are increasingly critical in the modern world. With these aims on mind, the present study employs survey data from Hubei Province in China to address two key research questions. Firstly, we use a multiple linear regression model to ascertain the influence of digital integration on the mental health of low-income populations. Furthermore, we investigate the disparities between urban and rural areas in digital integration and their impact on the mental health of low-income populations, considering the enduring urban–rural development gap in China. Secondly, we explore the mediating effect of social participation in the relationship between digital integration and the mental health of low-income populations.

## 4. Methods

### 4.1. Subjects and Sampling

The data used in this study were from the Comprehensive Social Survey of Urban and Rural Low-Income Populations in Hubei Province, China, 2022. Hubei, as a representative of the central provinces of China, exemplifies the socio-economic diversity observed across China’s urban and rural areas. It has undergone rapid urbanization, making it an important case study for examining the effects of urban–rural disparities in digital access and mental health. The present study employed a multi-stage stratified sampling technique, with six county administrative districts and 63 communities selected according to their level of economic development. A random sample of 20 residents aged over 18 years old was then selected from each urban and rural community, with a particular focus on social assistance recipients and individuals in low-income groups. The investigation covered sociodemographic information (age, gender, education level, marriage, etc.), household income and consumption, social protection, social participation, public services, digital integration, experience of hardship, and mental health. Participants were required to: (1) be at least 18 years of age; (2) can read and understand the Chinese questionnaire; and (3) volunteer to participate in the survey. A total of 1260 residents were invited to participate in the survey; 1177 fulfilled the study inclusion criteria and completed assessments, with an effective rate of 93.41%.

In alignment with the relative deprivation perspective, the present study delineates low-income populations in accordance with the national standard of income, specifically those whose income is less than 50 percent of the median per capita disposable income of Chinese residents in 2021 (equivalent to RMB 29,053). This income threshold is employed as the measure of relative deprivation. An individual or household is deemed to be relatively deprived if their income is below half of the median income level, thereby positioning them at a disadvantage compared to the broader population. This approach is consistent with the manner in which relative deprivation has been assessed in numerous developed countries, where economic inequality is frequently defined by income levels that are markedly inferior to the societal median [35]. The objective of this study is to contextualize low-income status within the national framework of China and in comparison to international standards, which will enable meaningful evaluation of economic disadvantage across different societal contexts. In this study, the term “low-income populations” is defined as urban and rural residents whose per capita household income is less than RMB 14,526.5. Following the processing of missing values and outliers for variables, a sample of 930 low-income populations was identified.

Table 1 shows a summarized overview of the demographics included in the analysis. Of the 930 respondents included in this sample, 53.87% of the respondents were male. Respondents were spread across various age groups, with the largest group being those aged 51–60 years (31.40%), followed by those aged 61–70 years (23.98%) and aged 41–50 years (19.35%), indicating that the majority of respondents were middle-aged or older. A significant proportion of the participants had elementary or junior high school education, with 33.87% having completed elementary school and 32.37% finishing junior high school, indicating that the majority of respondents had received primary and lower secondary education. A large proportion of participants were married (64.84%), with smaller groups being widowed (13.01%), divorced (11.29%), or single (10.86%). Only 24.73% of respondents considered themselves to be in good health, indicating that the overall health status of low-income populations was cause for concern. A majority of the participants lived in rural areas (60.54%), while 39.46% resided in urban areas.

### 4.2. Measures

#### 4.2.1. Dependent Variable: Mental Health

In alignment with the extant literature, the Self-Rating Anxiety Scale (SAS) and Self-Rating Depression Scale (SDS) were used to assess the mental health of participants [36], including representative six items (e.g., “I tend to get scared for no reason”, “I’m more nervous and anxious than usual”, “I tend to get psychologically upset or feel panicked”, “I feel emotionally frustrated and depressed”, “I feel like my life is meaningless”, “I feel like I’m useless”). Participants were asked to rate to what degree each statement applies to them. The measure was scored using a five-point Likert scale (1 = A great deal to 5 = Not at all). In the present study, Cronbach’s alpha was 0.815. The sum score of six items was used to represent the level of mental health, with higher scores indicating greater levels of mental health.

#### 4.2.2. Core Independent Variable: Digital Integration

In the present study, the term “digital integration” was employed to signify both the capacity to access the internet and the ability to utilize it for a range of purposes in daily life. Internet access was assessed via one item, ‘Do you have internet access in your home (mobile device, broadband, Wi-Fi)?’. A value of 1 was assigned to responses indicating “yes”, while a value of 0 was assigned to responses indicating “no”. The frequency of internet usage was gauged by inquiring of respondents as to the regularity with which they engaged in six specific online activities: communication and social interaction, leisure and entertainment, information seeking, education and learning, purchasing goods and services, and access to online services (such as online transportation or navigation applications). Respondents were asked to rate the extent to which each statement was applicable to them. The measure was scored using five-point Likert scale (1 = Never to 5 = Every day). In the present study, Cronbach’s alpha of the six items measuring internet usage was 0.852. The sum score of seven items including internet access and internet usage was used to represent the level of digital integration, with higher scores indicating greater levels of digital integration.

#### 4.2.3. Mediating Variable: Social Participation

The level of social participation was gauged by inquiring of respondents the frequency with which they engaged in four categories of offline activities: group physical exercise, leisure and recreation (chess and cards), social gatherings with family and friends, and live cultural appreciation. Respondents were asked to rate the extent to which each statement is applicable to them. The measure was scored using five-point Likert scale (1 = Never to 5 = Every day). In the present study, Cronbach’s alpha of the four items was 0.766. The sum score of four items was used to represent the level of social participation, with higher scores indicating greater levels of social participation.

#### 4.2.4. Control Variables

Participants were asked to state their age, gender, education level, marriage, self-rated health, residential area, and area dummy variables. In terms of assignment, gender was assigned as 1 for male and 0 for female. Age was a continuous variable, ranging from 18 to 101 years old. The value assigned to no education (illiterate) was 1, that assigned to having finished elementary school was 2, that assigned to having finished junior high school was 3, that assigned to having graduated from high school was 4, and that assigned to having graduated from college and above is 5. Marriage was divided into two types: married and unmarried (single/divorced/widowed), with 1 indicating married. Self-rated health was assigned as 1 for healthy and 0 for unhealthy (frail/chronic disease/serious illness/disability). Household income was defined as the per-capita income of the respondent’s household in 2021, and was a continuous variable. Household socio-economic status was a subjective assessment by respondents, for which respondents used a five-point Likert scale (1 = lower; 2 = lower middle; 3 = middle; 4 = upper middle; 5 = upper); the higher the score, the better the household’s socio-economic status. Residential area was assigned as 1 for urban respondents and 0 for rural respondents. Table 2 provides the descriptive statistics of each of the abovementioned variables.

### 4.3. Data Analyses

All analyses were conducted using Stata MP 17. Multiple linear regression analyses were used to explore the association between digital integration and mental health, as this method allows for the inclusion of multiple predictors while controlling for potential confounding variables. This approach was chosen due to its ability to assess the joint effects of several factors on mental health outcomes, assuming a continuous outcome variable. The OLS regression model is shown in Equation (1):

(1) $${mental\;health}_{i}=\alpha_{0}+\alpha_{1}{digital\;integration}_{i}+\delta_{1c}X_{i}+\varepsilon_{1i}$$

where ${mental\;health}_{i}$ is the dependent variable, ${digital\;integration}_{i}$ is the core independent variable. $X_{i}$ is a set of control variables, the estimated coefficient $\alpha_{1}$ is the coefficient of the digital integration effect on mental health, and $\varepsilon_{i}$ is a random disturbance term.

In addition, Equations (1)–(3) describe the mediating effect model developed in the present study, where ${social\;participation}_{i}$ reflects the mediating variables. The causal step regression method proposed by Baron and Kenny was used to test the mediating effect [37], as it allows for a step-by-step assessment of the relationships between independent variables, mediators, and dependent variables. Furthermore, to provide additional validation of the robustness of the mediating effect, the bootstrap method was employed. This approach provides additional validation by generating confidence intervals for the mediating effect, helping to ensure that the observed mediation is not due to sampling variability. In the present study, the bootstrap mediation test with 1000 repetitions of sampling was conducted using stata17 software.

(2) $${social\;participation}_{i}=\beta_{0}+\beta_{1}{digital\;integration}_{i}+\delta_{2c}X_{i}+\varepsilon_{2i}$$

(3) $${mental\;health}_{i}=\gamma_{0}+\gamma_{1}{digital\;integration}_{i}+\gamma_{2}{social\;participation}_{i}+\delta_{3c}X_{i}+\varepsilon_{3i}$$

## 5. Results

### 5.1. Digital Integration and Its Association with Mental Health

Table 3 presents the results of the multivariable linear regression analyses. The results of Model 1 demonstrated that digital integration had a statistically significant positive effect on the mental health of low-income populations, indicating that digital integration was an effective strategy for improving the mental health of low-income populations. Concerning the control variables and regional fixed effect in Model 2, digital integration was positively correlated with the mental health of low-income populations (B = 0.074, t = 3.11, *p* < 0.01). Briefly, a significantly higher level of digital integration by low-income populations in the digital age led to a higher level of mental health. Consistent with previous studies [38], our results showed that gender (B = 1.477, t = 5.50, *p* < 0.01), older age (B = 0.053, t = 4.16, *p* < 0.01), educational level (B = 0.317, t = 1.95, *p* < 0.1), self-rated health (B = 0.987, t = 3.74, *p* < 0.01), household income (B = 0.244, t = 1.86, *p* < 0.1), and household socio-economic status (B = 1.050, t = 5.96, *p* < 0.01) were independently and significantly associated with higher mental health level. Inconsistent with the findings of previous studies, marriage (B = −0.493, t = −1.78, *p* < 0.1) was independently and significantly associated with lower levels of mental health, which indicated that the prevalence of mental health issues was higher among married individuals in the low-income groups as compared to among their unmarried counterparts. This discrepancy may be attributed to the heightened livelihood pressures experienced by married individuals in low-income households. The impact of residential area on the mental health of low-income populations was not statistically significant.

### 5.2. Robustness Analysis

Given that the digital integration of low-income populations may not be randomly distributed, but rather a self-selection process determined by individual, household, community, and other characteristics, the direct inclusion of the low-income populations’ digital integration in the regression model may result in selectivity bias due to the presence of non-random sampling. To address the potential for selective bias and the limitation of traditional propensity score matching (PSM) models, which can only assess the net effect when the treatment variable is a dummy variable, the present study employed the generalized propensity score matching (GPSM) method, which is suitable for continuous variables, to estimate the net effect of digital integration on the mental health of low-income populations at varying treatment levels.

**Figure 1:**
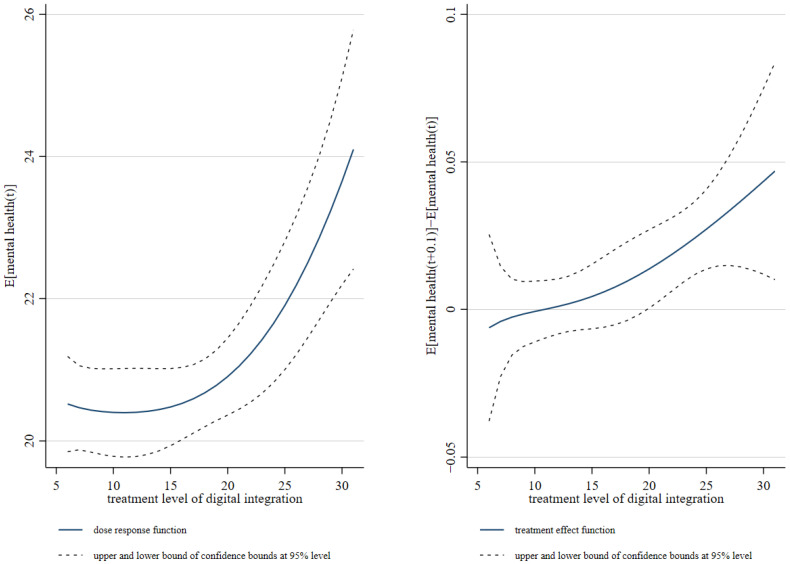
Impact of digital integration on the mental health of low-income populations.

In particular, we employed a methodology based on the estimation of the distribution of numerical incorporation to calculate the propensity score values and to match the samples. Given that the distribution of digital integration in the interval [6,31] is concentrated near the lower value, we attempted to subdivide the portion with smaller values of processing intensity and coarsely divide the portion with larger values. The values of processing intensity were selected as the 20th, 40th, and 60th percentiles, and the low-income populations samples were divided into four groups according to the values of processing intensity. Within each group, the samples were divided into five segments according to the value of the propensity score. Subsequently, the samples within each group were divided into five segments according to the values of the propensity score. To enhance the robustness of the results, a third-order polynomial was selected to model the relationship between digital integration and the mental health of low-income populations. In estimating the mean dose–response function and treatment effects, the present study set a step size of 1 and a total of 26 treatment intensity values, ranging from 6 to 31, in increments of 1. Figure 1 illustrates the correlation between the degree of digital integration and the mental health of low-income populations, as determined through the GPSM approach. In this instance, the left-hand panel presented the mean dose-response function, while the right-hand panel illustrated the impact of varying degrees of digital integration on the mental health of low-income populations (treatment effect). As illustrated in Figure 1, the overall impact of digital integration on the mental health of low-income populations was positive. An increase in the level of digital integration enhanced the mental health of low-income populations, which was consistent with the results of the benchmark regression above.

### 5.3. Digital Integration, Social Participation, and Their Association with Mental Health

The causal step regression method was used to test the mediating effect of social participation in the relationship between digital integration and mental health. As is shown in Table 4, it was consistent with the earlier estimation that digital integration had a significant positive effect on the mental health in Model 3. Digital integration (B = 0.079, t = 4.70, *p* < 0.01) was significantly associated with higher social participation in Model 4, indicating that the incorporation of digital technologies has been demonstrated to markedly enhance the level of social participation among economically disadvantaged populations. After controlling both digital integration and social participation, digital integration (B = 0.063, t = 2.56, *p* < 0.01) and social participation (B = 0.146, t = 2.79, *p* < 0.01) were positively associated with mental health, whereas the coefficient of digital integration decreased from 0.074 in Model 3 to 0.063 in Model 5. It can, therefore, be posited that social participation played a significant mediating role in the relationship between digital integration and the mental health of low-income populations. In other words, digital integration increased the social participation of low-income populations, which in turn affected their mental health.

In light of the regression results pertaining to the aforementioned mediation mechanism, the present study proceeded to undertake a further examination of the mediation effect through the utilization of the bootstrap test. The results of the bootstrap test, as presented in Table 5, indicated that the coefficient of the direct effect of digital integration on the mental health of low-income populations was 0.063, while the coefficient of the indirect effect of social participation on the mental health of low-income populations was 0.011. Notably, neither of these coefficients contained 0 within the 95% confidence interval, thereby passing the test. Accordingly, the results of the bootstrap test substantiated the mediating role of social participation.

### 5.4. Analysis of Urban–Rural Heterogeneity

The aforementioned results represented the mean effect observed across the entire sample. It should be noted that the differences between the various groups have not been taken into account in this analysis. In light of the enduring urban–rural development disparity in China, considerable discrepancies exist with regard to the extent of digital advancement between urban and rural regions. Consequently, the digital integration of low-income populations is contingent upon not only their individual digital literacy but also the digitalization level of the region in question. In order to analyze the heterogeneity of the mental health promotion effects of digital integration, the low-income populations were divided into urban and rural low-income groups based on area of residence.

As is shown in Table 6, the incorporation of digital technologies has been demonstrated to exert a considerable beneficial influence on the mental well-being of urban populations with limited financial resources. Conversely, the impact of digital integration on the mental health of rural populations with similar socioeconomic characteristics has not been found to reach statistical significance.

## 6. Discussion

The present study aimed to elucidate the intricate interrelationship between digital integration, social participation, and mental health among low-income populations in China. The findings provide new insights into how digital integration and social participation impact the mental well-being of low-income populations, particularly for individuals in different socio-economic settings and cultural contexts. Nevertheless, as the findings are not limited to China, this discussion has implications that extend beyond a domestic context. It compares findings with those of other low-income and developing countries and addresses the unique contributions to the literature, interventions, risks, and urban–rural disparities.

### 6.1. The Role of Digital Integration in Mental Health

The paper finds that digital integration is positively correlated with mental health outcomes, specifically relieving anxiety and depression, among low-income individuals. This implies that greater internet usage for constructive purposes may be linked to lower mental distress, which is consistent with some previous findings [39]. The idea that digital integration is associated with better mental health, particularly for low-income populations, might initially seem counterintuitive. Historically, the digital divide has been seen as a barrier that eliminates inequalities [18]. However, the results suggest that access to the internet can serve as a valuable tool for improving mental health in an era of extensive internet usage, particularly by enabling communication, access to information, and entertainment. For individuals who are financially disadvantaged, digital integration can offer a sense of connection to the outside world and reduce feelings of isolation, which are often linked to mental health challenges [40]. It also provides an avenue for learning and personal growth, helping to mitigate the stressors that come with economic hardship.

A comparison of this finding with those from other countries indicates that digital tools, including digital access and online mental health interventions, have the potential to enhance mental health outcomes by increasing access to care for underserved populations [28]. Nevertheless, in countries such as India or South Africa, while digital health interventions have demonstrated potential, challenges including low digital literacy, unreliable internet access, and financial limitations have impeded their widespread effectiveness [41,42]. These findings are consistent with the results of this study, which highlights how digital integration may enhance mental health in low-income Chinese populations, but only when certain conditions, such as reliable internet access and higher digital literacy, are met. Although digital integration offers clear benefits in high-income settings, the findings of this study indicate that in China’s low-income populations, particularly in rural areas, there is still a significant gap in digital access and utilization, which can limit the overall impact of digital tools. The structural barriers to digital integration observed in China are reflected in numerous developing countries, where digital technologies are regarded as a means of mental health support but are frequently inaccessible due to infrastructural constraints [7].

### 6.2. The Mediating Role of Social Participation

A notable finding in the present study is that social participation acts as a significant mediator between digital integration and the mental health of low-income populations. It suggests that the positive mental health outcomes linked to digital integration may be facilitated through enhanced social interactions and engagement in offline social activities. It aligns with existing literature suggesting that social support is a critical factor in mental health, particularly for low-income individuals [43]. This use of digital tools enables individuals to connect with others, access essential services, and engage in activities that support social participation, all of which contribute to better mental health outcomes. For instance, digital tools can help individuals find local social groups, maintain relationships with distant family members and friends, and access community resources, which all support social engagement and reduce isolation—a significant contributor to anxiety and depression [44].

This study also highlights the importance of offline social participation. Even in the context of increasing digital connectivity, face-to-face interaction remains a crucial component of mental well-being. Offline activities such as group physical exercise or cultural appreciation provide tangible benefits in terms of physical and mental health [45]. These activities promote a sense of belonging, community, and personal fulfillment, which cannot be fully replaced by online interactions. It is also worth noting that the activities listed in the paper reflect a broad range of engagement, from physical and recreational activities to cultural appreciation, which may appeal to diverse segments of the low-income populations. The broad scope of social participation may be particularly relevant for fostering a sense of purpose and connection in populations that are otherwise marginalized. Interestingly, digital tools can make offline participation easier for individuals, especially those who are isolated or face geographical or mobility challenges [46]. For example, the usage of online platforms can help individuals locate local events or groups they might otherwise be unaware of, or coordinate in-person meetups that foster social ties and community involvement.

Nevertheless, while our findings are encouraging, it is essential to recognize the complexities of the relationship between digital integration, social participation, and mental health. While digital tools can enhance social participation, they may not universally address the underlying structural and systemic barriers that contribute to poor mental health in low-income populations [16], such as inadequate housing, unemployment, or limited access to healthcare. It is imperative to consider digital integration as a crucial component of a comprehensive strategy for promoting mental health and well-being. Additionally, it is crucial to consider the potential risks associated with digital engagement, such as cyberbullying, misinformation, or digital overload, which could have negative consequences on mental health [33]. Further research is needed to explore these risks and to identify strategies for mitigating them.

### 6.3. Urban–Rural Disparities and the Impact of Digital Integration

The present study demonstrates a notable discrepancy in the influence of digital integration on the mental well-being of low-income individuals, contingent on their geographical location, whether urban or rural. Specifically, digital integration has a significant positive impact on the mental health of urban low-income populations, whereas it does not have a statistically significant effect on rural populations, which is also reflected in other populations [47]. The finding suggests that the benefits of digital integration are not distributed evenly across geographical areas, and there may be additional factors, beyond just internet access, that influence how digital tools are used in different contexts. One possible explanation for this divergence is the difference in internet infrastructure and access between urban and rural areas. Urban areas are generally better equipped with reliable and high-speed internet, which may enhance the ability of individuals to fully engage with digital resources, such as social platforms, educational tools, and health services [48]. In contrast, rural areas in China, as well as in countries like India and Brazil, may face more significant barriers to digital integration, such as unreliable internet access, lower levels of digital literacy, or fewer opportunities to leverage online services for personal growth, education, or social interaction [41]. These barriers can hinder the full potential of digital integration to improve mental health in rural areas.

However, the differences between urban and rural areas go beyond just technical limitations. Cultural and social factors in rural communities may also play a role in the limited impact of digital integration on mental health. Rural areas are frequently distinguished by tight-knit communities and strong local networks, where face-to-face interactions and family support systems are more prevalent [49]. This strong social cohesion can diminish the necessity for digital tools, as individuals in rural areas are more likely to receive emotional support and maintain social connections through traditional, offline channels. The reliance on local community values and personal relationships in rural settings may, thus, render the need for digital engagement less pressing compared to urban environments, where individuals may experience greater social isolation or urban stressors that make digital tools a crucial part of their social fabric [48]. It should be noted, however, that rural populations may also face unique cultural attitudes towards technology that can influence their utilization of digital tools. In some rural areas, there may be a preference for face-to-face communication over digital interaction [50], or a skepticism towards technology’s role in social life. This could limit the extent to which digital tools are embraced as effective resources for mental health support.

As rural areas continue to undergo urbanization and demographic shifts, the strength of traditional social networks may be eroded, while the necessity for digital integration is likely to intensify. The disintegration of local, face-to-face support systems in rural areas could highlight the importance of digital tools in maintaining social connections and accessing essential services, such as healthcare, education, and mental health support. As such, strengthening digital infrastructure and fostering digital literacy in rural areas may become increasingly important in ensuring that rural populations are able to participate fully in the digital age and benefit from the mental health advantages associated with digital integration [51]. Conversely, urban populations may be more reliant on digital tools for a range of purposes, including social interaction, information seeking, and entertainment, especially given the higher levels of urban stressors, such as social isolation, competitive work environments, and urban alienation. These stressors make offline social participation more challenging, and digital tools can serve as a vital lifeline to improve mental well-being in urban settings, particularly for those facing limited offline engagement opportunities. It can be reasonably deduced that digital integration may prove to be a pivotal factor in the improvement of mental health among low-income urban populations.

### 6.4. Policy and Practical Implications

Our findings indicate the potential for several practical and policy recommendations to facilitate the reduction of the digital divide and enhance mental health outcomes in low-income populations. Firstly, policymakers should expand digital access in rural areas. Concrete actions could include government-funded initiatives to construct broadband infrastructure in underserved rural areas, leveraging public–private partnerships to facilitate affordable internet access for low-income households. Additionally, social enterprises or NGOs could collaborate with local governments to distribute affordable or subsidized digital devices, such as smartphones and tablets, to residents in rural areas. It is imperative that these infrastructural deficiencies be addressed if rural residents are to be enabled to benefit from digital integration. In countries such as India, government programs such as the BharatNet initiative have been instrumental in increasing digital connectivity in rural areas, and could serve as a model for low-income or developing countries. Secondly, in both urban and rural populations, especially among low-income groups, targeted training programs could be established in collaboration with community centers or local schools, offering free or subsidized digital literacy workshops that equip individuals with the skills needed to effectively use digital technologies for mental health support, social participation, and economic opportunities. Thirdly, initiatives designed to encourage social participation in both online and offline contexts should be incorporated into digital integration policies. For example, creating community-based digital hubs or virtual support groups tailored to low-income populations can enhance the mediating role of social participation in mental health. Additionally, in rural areas, digital technologies should be complemented with community-driven approaches that leverage existing social networks and cultural practices. Locally relevant digital content and services should be developed to ensure that rural residents can derive tangible benefits from digital integration. Fourthly, it is imperative that policymakers address the potential risks associated with digital engagement, including cyberbullying, misinformation, and digital overload, which could exacerbate mental health issues. It is, therefore, recommended that national mental health strategies incorporate policies to regulate online spaces, provide digital mental health education, and offer support to individuals experiencing distress as a result of digital engagement.

### 6.5. Strengths and Limitations

Our study had several strengths. Firstly, the paper employs a comprehensive framework that incorporates both online and offline factors in order to gain a full understanding of the factors that contribute to mental health outcomes. By examining not just the role of digital integration but also the mediating effect of social participation, the study provides a more detailed and sophisticated understanding of the pathways through which digital technologies can impact well-being. Secondly, the paper’s focus on differentiating between urban and rural populations represents a significant strength. The comparison permits the investigation of disparities in the impact of digital integration on mental health, which can facilitate the development of targeted interventions that address the distinctive requirements of rural populations. Thirdly, the study contributes to the expanding corpus of literature examining the nexus between digital technology, social participation, and mental health. By focusing on low-income populations, it addresses a gap in research, which has previously overlooked the most vulnerable groups, contributing to a more inclusive understanding of digital mental health interventions.

There were some limitations in this study. The initial limitation is the use of a cross-sectional survey, limiting our ability to make statements about causal relationships. A further limitation is that the levels of psychological impact, anxiety, and depression were self-reported, which may not always align with objective assessment by mental health professionals. Finally, while the present study focused on the positive aspects of digital integration, we did not examine the potential negative impacts, such as the risks of digital overload, exposure to harmful content, or cyberbullying. These factors could affect mental health and social participation, and future studies should investigate both the positive and negative outcomes of digital engagement.

## 7. Conclusions

Despite the above limitations, the findings of this study highlight the complex relationship between digital integration, social participation, and mental health. While digital access appears to have a positive correlation with mental well-being in low-income populations, this effect is mediated by social participation, which emphasizes the importance of offline activities in fostering mental health. The differential impact of digital integration on urban versus rural populations underscores the necessity for targeted interventions that take into account regional disparities in internet access and social support structures. In light of these findings, policymakers, healthcare providers, and community organizations should work together to ensure that marginalized populations can access and benefit from digital technologies in ways that promote their mental health and social well-being.

## Figures and Tables

**Table 1:** Demographic characteristics.

	N	%
Gender		
Male	501	53.87
Female	429	46.13
Age		
18–40 years	84	9.03
41–50 years	180	19.35
51–60 years	292	31.40
61–70 years	223	23.98
>71 years	151	16.24
Education level		
No education (illiterate)	134	14.41
Elementary school	315	33.87
Junior high school	301	32.37
High school	146	15.69
College and above	34	3.66
Marriage		
Single	101	10.86
Married	603	64.84
Divorced	105	11.29
Widowed	121	13.01
Self-rated health		
Health	230	24.73
Frail	129	13.87
Chronic disease	303	32.58
Serious illness	156	16.77
Disability	112	12.04
Residential area		
Urban areas	367	39.46
Rural areas	563	60.54

**Table 2:** Descriptive statistics of the variables.

Variable Name	Mean	SD	Min	Max
Mental health	16.132	3.468	6	30
Digital integration	13.076	6.918	6	31
Social participation	7.537	2.631	4	20
Gender	0.539	0.499	0	1
Age	57.799	12.513	18	101
Education level	2.603	1.031	1	5
Marriage	0.648	0.478	0	1
Self-rated health	0.386	0.487	0	1
Household income	6323.202	3526.848	0	14,520
Household socio-economic status	1.872	0.806	1	5
Residential area	0.395	0.189	0	1

**Table 3:** Association between digital integration and the mental health of low-income populations.

Variables	Model 1		Model 2	
	B	SE	B	SE
Digital integration	0.072 ***	0.020	0.074 ***	0.024
Gender			1.477 ***	0.269
Age			0.053 ***	0.013
Education level			0.317 *	0.162
Marriage			−0.493 *	0.277
Self-rated health			0.987 ***	0.264
Household income			0.244 *	0.131
Household socio-economic status			1.050 ***	0.176
Residential area			0.140	0.359
Regional fixed effect	Uncontrolled		Controlled	
Constant	19.835 ***	0.300	11.582 ***	1.843
Samples	930		930	
F	13.153		14.689	
Adj R-squared	0.013		0.149	

Notes: * *p* < 0.1, *** *p* < 0.01. SE is robust standard error. In order to reduce heteroskedasticity in the dataset and enhance the precision of the model, the household income variable was transformed using the logarithm function.

**Table 4:** The mediating effect of social participation on mental health.

Variables	Model 3		Model 4		Model 5	
	Mental Health		Social Participation		Mental Health	
	B	SE	B	SE	B	SE
Digital integration	0.074 ***	0.024	0.079 ***	0.017	0.063 ***	0.025
Social participation	——	——	——	——	0.146 ***	0.052
Control variables	Controlled		Controlled		Controlled	
Regional fixed effect	Controlled		Controlled		Controlled	
Constant	10.789 ***	1.486	4.457 ***	1.040	10.137 ***	1.475
Samples	930		930		930	
F	14.689		9.225		15.864	
Adj R-squared	0.149		0.125		0.156	

Notes: *** *p* < 0.01, and standard error is robust standard error; the control variables were consistent with baseline regression.

**Table 5:** Results of the mediation mechanism test based on the bootstrap methodology.

	Observed Coefficient	Bootstrap Standard Error	Confidence Bounds at 95% Level	
			Lower Bond	Upper Bond
Direct effect	0.063	0.025	0.016	0.115
Indirect effect	0.011	0.005	0.003	0.022

**Table 6:** The regression results of the subgroup samples.

Variables	Model 6		Model 7	
	Urban Area		Rural Area	
	B	SE	B	SE
Digital integration	0.119 ***	0.038	0.045	0.031
Control variables	Controlled		Controlled	
Regional fixed effect	Controlled		Controlled	
Constant	10.003 ***	2.156	12.454 ***	2.089
Samples	367		563	
F	9.104		11.494	
Adj R-squared	0.172		0.157	

Notes: *** *p* < 0.01, and standard error is robust standard error; the control variables were consistent with baseline regression.

## Data Availability

The data presented in this study are available on request from the corresponding author (Beihai Tian).
